# A potential link between fibroblast growth factor-23 and the progression of AKI to CKD

**DOI:** 10.1186/s12882-023-03125-1

**Published:** 2023-04-04

**Authors:** Yinghui Lu, Shutian Xu, Rong Tang, Cui Han, Chunxia Zheng

**Affiliations:** Jinling Hospital, National Clinical Research Center of Kidney Diseases, Nanjing University School of Medicine, Nanjing, China

**Keywords:** AKI – CKD, Renal fibrosis, FGF-23

## Abstract

**Background:**

Patients who recover from acute kidney injury (AKI) have a 25% increase in the risk of chronic kidney disease (CKD) and a 50% increase in mortality after a follow-up of approximately 10 years. Circulating FGF-23 increases significantly early in the development of AKI, is significantly elevated in patients with CKD and has become a major biomarker of poor clinical prognosis in CKD. However, the potential link between fibroblast growth factor-23 levels and the progression of AKI to CKD remains unclear.

**Method:**

Serum FGF-23 levels in AKI patients and ischaemia‒reperfusion injury (IRI) mice were detected with ELISA. Cultured HK2 cells were incubated with FGF-23 and PD173074, a blocker of FGFR, and then TGFβ/Smad and Wnt/β-catenin were examined with immunofluorescence and immunoblotting. Quantitative real-time polymerase chain reaction was used to detect the expression of COL1A1 and COL4A1. Histologic staining confirmed renal fibrosis.

**Results:**

The level of serum FGF-23 was significantly different between AKI patients and healthy controls (*P < 0.01*). Moreover, serum FGF-23 levels in the CKD progression group were significantly higher than those in the non-CKD progression group of AKI patients (*P < 0.01*). In the AKI-CKD mouse model, serum FGF-23 levels were increased, and renal fibrosis occurred; moreover, the protein expression of β-catenin and p-Smad3 was upregulated. PD173074 downregulated the expression of β-catenin and p-Smad3 and reduced fibrosis in both mice and HK2 cells.

**Conclusion:**

The increase in FGF-23 may be associated with the progression of AKI to CKD and may mediate renal fibrosis via TGF-β and Wnt/β-catenin activation.

**Supplementary Information:**

The online version contains supplementary material available at 10.1186/s12882-023-03125-1.

## Background

Acute kidney injury (AKI) is a common clinical problem in hospitalized patients worldwide, and it is one of the most common acute and critical illnesses in clinical departments. AKI not only affects the patient survival rate, long-term prognosis and quality of life but also places a huge economic burden on the patient’s family and society. AKI confers formidable morbidity and mortality in its acute phase, and among survivors of AKI, the long-term outcomes are far from benign. Patients who recover from AKI have a 25% increase in the risk of chronic kidney disease (CKD) and a 50% increase in mortality after a follow-up of approximately 10 years [1–3]. Although clinical observations describe a clear association, the underlying mechanism of AKI progression to CKD needs to be explored.

Bone-derived fibroblast growth factor-23 (FGF-23) is an important endocrine regulator of mineral homeostasis, and its effects are transduced by cognate FGF receptor (FGFR)1-α-Klotho complexes [3, 4]. Circulating FGF-23 levels increase precipitously in patients with kidney diseases and indicate worse renal and cardiovascular outcomes [5, 6]. FGF-23 levels increase early in CKD [7], predominantly as intact protein, and generally precede changes in other mineral metabolites [8]. Similarly, circulating FGF-23 concentrations increase rapidly in AKI, also preceding changes in other mineral markers and conventional measures of renal function [9].

Renal fibrosis is the pathological basis of CKD [10]. The canonical TGF-β1/smad3 signalling pathway mediates the transdifferentiation of renal tubular epithelial cells and plays an important role in the progression of renal fibrosis [11]. Animal studies of ureteral obstruction (UUO) have shown that tubule-derived FGF-23 can enhance the activity of myofibroblasts during AKI, possibly promoting the signalling cascade of renal fibrosis by activating TGF-β channels. [12, 13] The Wnt/β-catenin signalling pathway is closely related to the occurrence and development of renal interstitial fibrosis. Persistent activation of the Wnt/β-catenin pathway plays an important role in promoting the development of AKI to CKD [14].

In this study, we examined whether the increase in circulating FGF-23 was associated with CKD development after AKI. Then, we explored the potential molecular mechanism of FGF-23 in the progression of AKI to CKD. Our study aimed to uncover a novel mechanism of the progression of AKI to CKD and provides a potential therapeutic target for preventing and improving the prognosis of AKI.

## Materials and methods

### ***AKI patients***

We selected 94 AKI patients who were admitted to the Nephrology Intensive Care Unit (ICU) of the National Kidney Disease Clinical Research Center and included 60 healthy humans in the same period as the control group. AKI diagnosis and staging criteria were based on the AKI guidelines of the Kidney Disease Improvement Global Prognosis Organization (KDIGO) [15]. The diagnostic criteria for CKD were a basal eGFR of less than 60 ml/(min 1.73 m^2^) and a duration of more than 3 months.

### ***Animals***

C57BL/6 mice (male, 12 weeks old) were purchased from Gem Pharmatech at Nanjing. The mice were housed individually at 23 °C with a 12:12-h light-dark cycle and were maintained on water and food.

### ***Surgery protocols***

Briefly, the mice were anaesthetized with pentobarbital (50 mg/ml, i.p.), and the body temperature was maintained at 36.8–37.5 °C during surgery with a temperature-controlled operating table. The renal pedicle was carefully dissected and clamped with a silver clip for 35 min. After releasing the clip, the wound was sutured. Sham-operated animals without clamping served as controls.

### ***Treatment with PD173074***

The pan-FGF receptor blocker PD173074 (Sigma‒Aldrich, Inc.) was dissolved in PBS. The mice (sham and IRI, twelve-week-old male C57BL/6 mice) were intraperitoneally injected with PBS or PD173074 (1 mg/kg) once daily for the same duration. After 2 weeks, the animals were sacrificed, and blood and kidney tissues were collected.

### ***Serum biochemistry***

Blood samples (~ 200 µl/each) were centrifuged at 3,000 rpm for 10 min at 4 °C to separate the serum (~ 100 µl/each). Blood urea nitrogen and creatinine were measured by Servicebio (https://www.servicebio.cn/).

### ***FGF23 concentration assay***

The active intact FGF23 (iFGF23) enzyme-linked immunosorbent assay (ELISA) kit (catalog no. CY-4000; Kainos Laboratories, Tokyo, Japan) was used. It is a two-point ELISA kit for the determination of iFGF23 in serum.

### ***Morphological analysis of mouse tissues***

After blood collection, the animals were sacrificed. The kidneys were isolated and prepared for molecular and histological analyses. Kidneys tissues were stained with Masson dye to observe fibrosis.

### ***HK2 cell culture and treatments***

The HK2 cell line was purchased from ATCC, and the cells were cultured in F12 (Gibco, Inc.) supplemented with 10% foetal bovine serum (Gibco, Inc.) in an atmosphere of 5% (v/v) CO_2_ in air at 37 °C. The cells were treated with 25 ng/ml FGF-23 (Research & Diagnostics Systems, Inc.).

### ***Immunofluorescence***

After being treated, cultured HK2 cells were fixed in 4% paraformaldehyde and permeabilized with 0.1% Triton X-100 in PBS, followed by blocking with 5% goat serum in PBS. Rabbit monoclonal antibodies against p-smad3 (catalogue C25A9; Cell Signalling Technologies, Inc.) were used at 1:1000. A rabbit monoclonal antibody against β-catenin (catalogue 610,154; BD Biosciences, Inc.) was used at 1:2000. Cy3-conjugated goat anti-mouse (catalogue A0521; Beyotime Biotechnology, Inc.) was used as a secondary antibody at 1:500. To visualize nuclei, the fixed cells were incubated with DAPI (400 ng/mL in PBS) for 10 min. Immunofluorescence images were taken with a DM5000B microscope (Leica). The myocyte cross-sectional area was measured by ImageJ software (http://rsbweb.nih.gov/ij/).

### ***RNA extraction and quantification.***

Cultured HK2 cells and mouse kidney tissues were subjected to total RNA extraction with an isolation kit (Thermo Fisher Scientific, Inc.). In brief, this extraction method is based on the ability of glass fibers to bind nucleic acids in concentrated chaotropic salt solutions. Samples are disrupted in a typical high concentration guanidinium salt solution that simultaneously lyses cells and inactivates endogenous RNases. The lysate is diluted with an ethanol solution to make the RNA competent for binding to the glass fiber filter in the RNAqueous Filter Cartridge. This solution is passed through the filter pad where RNA binds and most other cellular contents flow through. The Filter Cartridge is washed 3 times to remove contaminants, and the RNA is eluted in a very low ionic strength solution. Reverse transcription of 300 ng of RNA to cDNA using the Reverse Transcription Kit (Takara Biomedical Technology (Beijing) Co., Ltd.). 2 reverse transcription primers, Random 6 mers and Oligo dT Primer, are included in the kit to synthesize cDNA suitable for Real Time PCR. mRNA sample was quantified by qPCR using a kit from TaKaRa (TaKaRa Bio, Inc.). The primers used are summarized in Table 1. For Q-PCR experiments, annealing temperature is set at 55–60 °C. Relative mRNA expression was evaluated with the 2^–ΔΔCT^ method using 18 S for normalization.

**Table 1:** The primers used in this study

	Forward sequence (5’→3’)	Reverse sequence (5’→3’)
COL1A1	GCTCCTCTTAGGGGCCACT	ATTGGGGACCCTTAGGCCAT
COL4A1	TCCGGGAGAGATTGGTTTCC	CTGGCCTATAAGCCCTGGT
18s	TTTCTCGATTCCGTGGGTGG	AGCATGCCAGAGTCTCGTTC

### ***Protein extraction and Western blot analysis***

Proteins were extracted from kidney tissues with RIPA buffer (Beyotime Biotechnology, Inc.) according to the manufacturer’s instructions. Antibodies against GAPDH (catalogue BS65529; Bioworld Technology, Inc.), α-SMA (catalogue ab5694; Abcam, Inc.), β-catenin (catalogue 610,154; BD Biosciences, Inc.) and p-smad3 (catalogue C25A9; Cell Signalling Technologies, Inc.), E-cad (catalogue 20874-1-AP; Proteintech, Inc.), and NGAL (catalogue ab216462; Abcam, Inc.) were used as primary antibodies, and horseradish peroxidase-conjugated goat anti-rabbit or anti-mouse (Beyotime Biotechnology, Inc.) secondary antibodies were used. Image analysis with image J software.

### ***Statistical analysis***

The data were tested for normal distribution. If the measures were normally distributed, they were expressed as mean ± SD, and the t-test was used for comparison between two groups, and Pearson correlation was used for correlation analysis. The Kruskal-Wallis test was used for comparison between two or more groups, and the Spearman’s rank correlation test was used for correlation analysis. The chi-square test was used for comparison of the sex and diabetes ratio. The correlation between AKI patients and FGF-23 was analysed by logistic regression analysis. *P* < 0.05 was considered statistically significant.

## Results

### FGF-23 is expressed at increased levels in AKI patients progressing to CKD

**Fig. 1:**
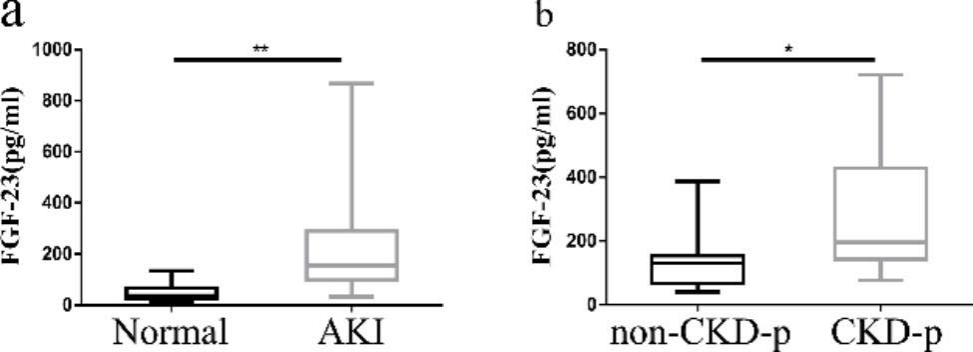
Serum FGF-23 levels in AKI patients. (a) Serum FGF-23 levels in AKI patients and healthy controls. (b) Serum FGF-23 levels of AKI patients in the CKD progression group and non-CKD progression group. non-CKD-P: non-CKD progression. CKD-p: CKD progression. (*P < 0.05; **P < 0.01)

There were 94 AKI patients were enrolled. The baseline characteristics of the patients are shown in Tables 2 and 3. The levels of serum FGF-23 were significantly different between AKI patients and healthy controls (152.65 (92.18, 293.90) versus 37.62 (26.40, 64.01) pg/ml pg/ml, *P* < 0.01, Fig. 1a). These AKI patients were followed up for three months after discharge to observe their progression to CKD. Eight of these patients lost follow-up at the three months. These AKI patients were divided into a CKD progression group (45 cases) and a non-CKD progression group (41 cases). Serum FGF-23 levels in the CKD progression group were significantly higher than those in the non-CKD progression group (189.05 (151.59, 282.87) pg/ml versus 230.60 (66.58, 152.68) pg/ml, *P* < 0.05, Fig. 1b). Collectively, these results indicated that AKI patients had a high level of circulating FGF-23, and higher FGF-23 levels were found in those patients that progressed from AKI to CKD.

**Table 2:** Characteristics of the AKI patients and healthy controls

	AKI (n = 94)	Controls (n = 60)	*P*
Sex (male/female)	52/42	32/28	> 0.05
Age	47.00 ± 16.90	46.65 ± 13.15	> 0.05
eGFR	8.44(5.23–12.49)	105.76(94.58-113.85)	< 0.001

Note: *P* < 0.05 was considered statistically significant. eGFR: estimated glomerular filtration rate

**Table 3:** Characteristics of the AKI patients at baseline

	AKI (n = 94)		
male n (%)		52 (55.3)	
Age		47.00 ± 16.90	
Body Mass Index		23.20 (20.00, 25.20)	
Systolic blood pressure		140.61 ± 21.87	
Diastolic blood pressure		83.27 ± 14.10	
Diabetes n (%)		10(10.6)	
C-reactive protein (mg/L)		21.60 (7.80, 94.00)	
IL-6 (ng/L)		22.01 (11.90, 42.87)	
Serum creatinine (mg/dl)		6.17 (4.69, 9.88)	
Blood urea nitrogen (mg/dl)		68.38 ± 31.13	

*P* < 0.05 was considered statistically significant. eGFR: estimated glomerular filtration rate.

Patients were divided into two groups based on FGF-23 levels (< 53.8 pg/ml and ≥ 53.8 pg/ml), and we found that the higher group had poorer renal recovery at discharge than the lower group (*P* < 0.05, Table 4). We performed logistic regression analysis and found that serum levels of FGF-23 were associated with AKI (OR = 1.044, *P < 0.001*) (Supplementary Table 1), also related with AKI progression to CKD (OR = 1.018, *P = 0.018*) (Supplementary Table 2).

**Table 4:** The correlation between FGF-23 level and AKI post CKD

FGF-23(pg/ml)	non-CKD-p(n = 41)	CKD-p(n = 45)	*P*
< 53.8(n = 15)	11(73.4%)	4(26.7%)	0.029
≥ 53.8(n = 71)	30(42.2%)	41(57.3%)	

Note: non-CKD-p: non-CKD progression. CKD-p: CKD progression. AKI post CKD: AKI progresses to CKD. *P* < 0.05 was considered statistically significant

### FGF-23 exhibits a sustained increase in the AKI-CKD mouse model

**Fig. 2:**
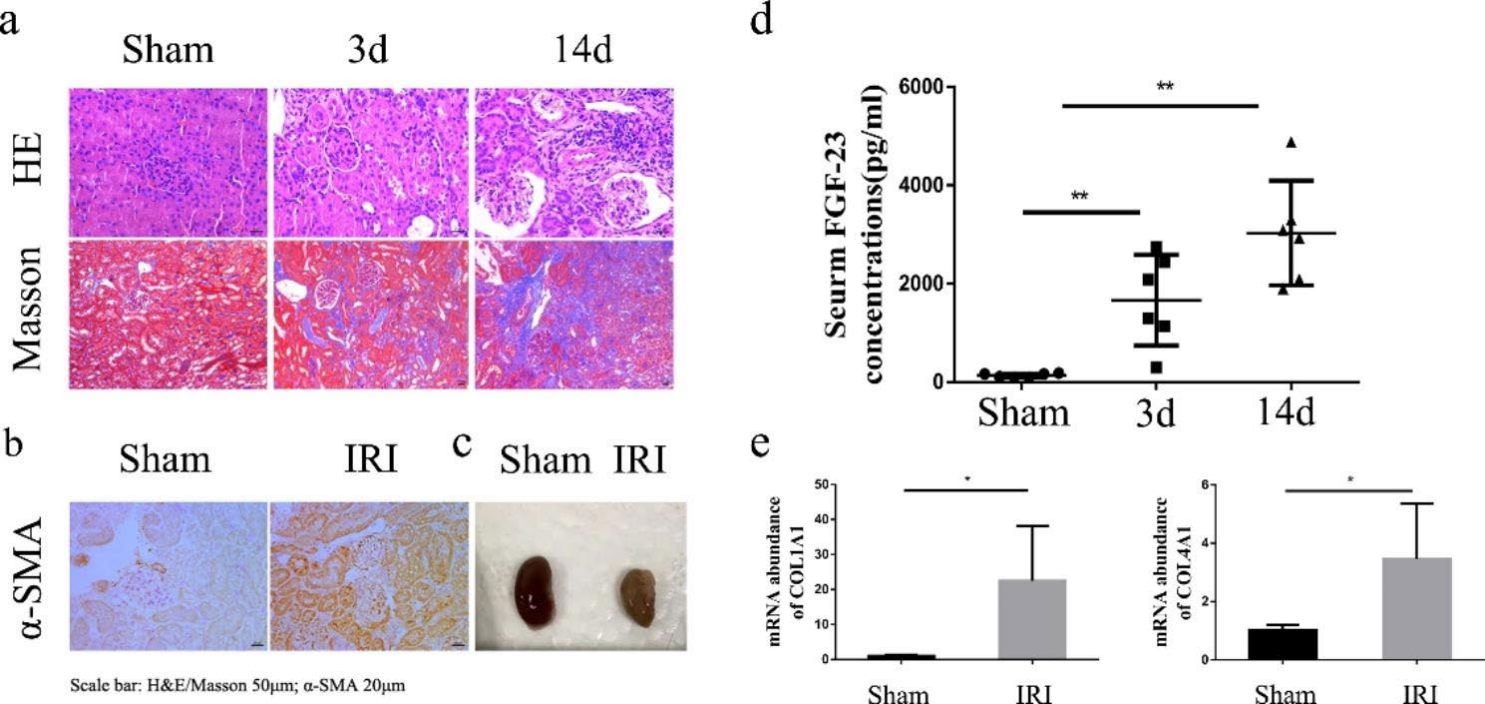
Renal fibrosis occurred in IRI mice with increased FGF-23 levels. (a) Representative photomicrographs of haematoxylin and eosin (H&E)- and Masson’s trichrome-stained kidney sections of sham and IRI mice. (b) Representative photomicrographs of immunochemical staining of kidney sections from sham and IRI mice. (c) Representative gross appearance of kidneys in the indicated groups. (d) Average concentrations of serum FGF-23 in sham and IRI mice (mean ± SD; n = 6 mice per group; *P < 0.05). (e) Renal expression of COL1A1 and COL4A1 in sham and IRI mice was assayed by Q-PCR (mean ± SD; n = 6 mice per group; *P < 0.05; **P < 0.01)

We generated a bilateral ischaemia‒reperfusion injury (Bi-IRI) mouse model. The bilateral kidneys of the mice were reperfused after 35 min of ischaemia. Kidney histologic staining revealed increasing pathologic changes including acute inflammatory infiltration, brush edge detachment, tubular atrophy and interstitial fibrosis 14 days after IRI (Fig. 2a). The epithelial-mesenchymal transition marker α-smooth muscle actin (α-SMA) was markedly induced at 14 days after IRI, as evidenced by immunofluorescent staining (Fig. 2b). At 14 days post-AKI, IRI caused obvious kidney shrinkage (Fig. 2c). The serum levels of FGF-23 were increased after 3 days of IRI, followed by sustained elevation until 14 days of IRI (Fig. 2d). Moreover, we found that COL1A1 and COL4A1 levels in kidney tissues were upregulated in IRI mice (Fig. 2e). These results suggested that two weeks after surgery, these mice showed markedly high expression of FGF-23 and obvious fibrosis. Consistent with the previous literature [16], renal fibrosis at 14 days post-AKI may be the precursor of CKD. In the next experiment, we used a mouse model 14 days after Bi-IRI.

### FGF-23 promotes profibrotic cellular signalling in HK2 cells

**Fig. 3:**
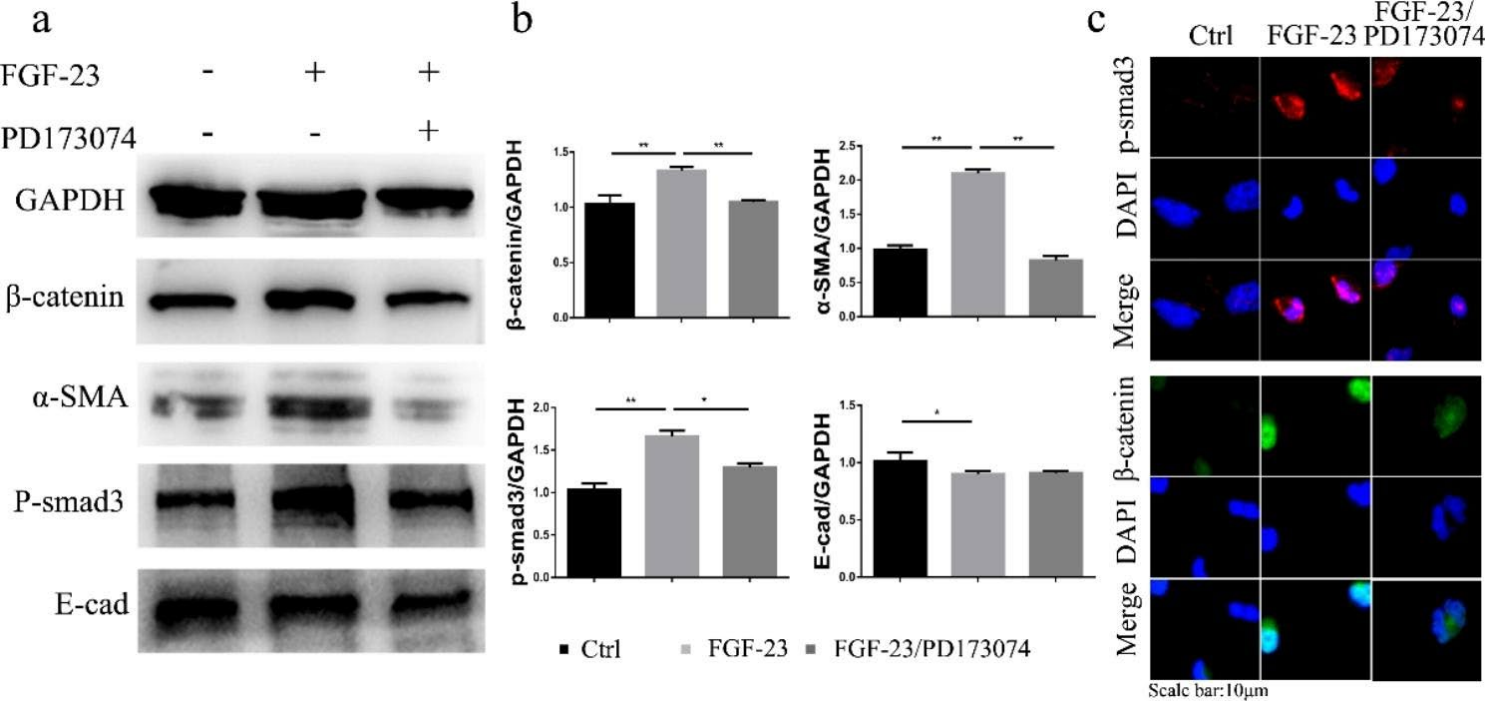
Fibrosis and nucleation of β-catenin/p-smad3 in HK2 cells after FGF-23 stimulation. (a) Expression of E-cadherin (E-cad), α-smooth muscle actin (α-SMA), β-catenin, and phosphorylated Smad3 in control, FGF-23-treated and FGF-23/PD173074-treated HK-2 cells, as determined by Western blotting. (b) Quantification of the data in (a). (*P < 0.05; **P < 0.01). (c) Representative immunofluorescent staining of β-catenin and phosphorylated Smad3 in HK2 cells treated with FGF-23 in the presence or absence of PD173074 for 24 h. The cells were also stained with DAPI (middle panel) and merged with P-Smad3/β-catenin images (lower panel). The original blue nuclear DAPI staining was converted to blue so that the green nuclear β-catenin and phosphorylated Smad3 staining was shown in red or green on merged images for easy recognition

To explore the molecular mechanism by which the increase in FGF-23 promotes renal fibrosis, we used renal tubular HK2 cells to observe the changes in response to FGF-23 and FGFR inhibitors. FGF-23 markedly reduced the epithelial marker E-cadherin (E-cad) and increased the myofibroblast marker α-SMA (Fig. 3a, b). Then, we examined the TGFβ/Smad and Wnt/β-catenin signalling pathways, which are the major profibrogenic pathways that are causatively related to renal fibrogenesis (Fig. 3a, b). FGF-23 activated Smad3 phosphorylation and β-catenin and led to their nuclear translocation in HK2 cells (Fig. 3c). In contrast, the pan-FGFR inhibitor PD173074 reduced Smad3 phosphorylation and β-catenin expression, as well as their nuclear translocation (Fig. 3c). Thus, we speculate that FGF-23 may promote renal fibrosis via TGFβ/Smad and Wnt/β-catenin signalling.

### FGFR inhibition promotes kidney recovery and reduces renal fibrosis in AKI-CKD mice

**Fig. 4:**
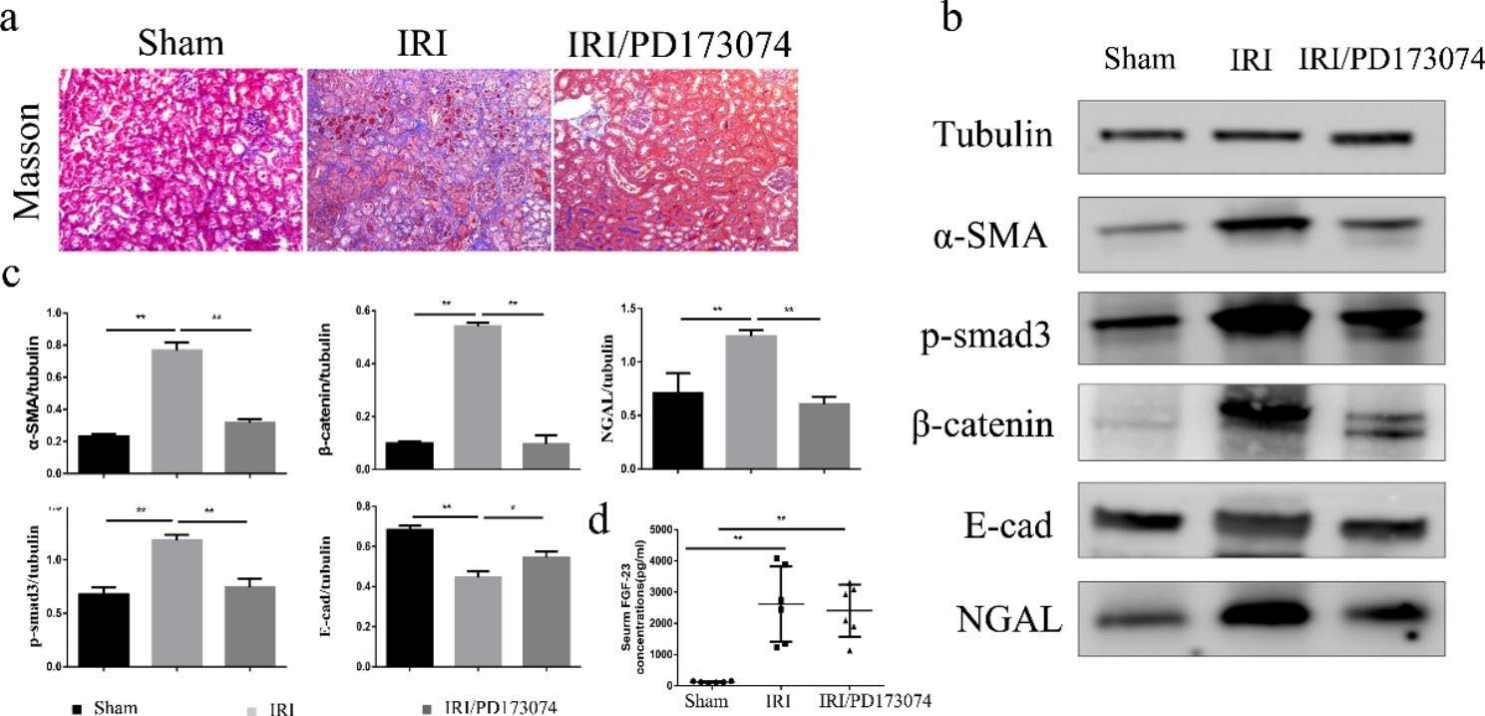
PD173074 reduced renal fibrosis in AKI-CKD mice. (a) Representative photomicrographs of Masson’s trichrome-stained kidney sections from control, IRI-treated and IRI/PD173074-treated mice. (b) Kidney expression of E-cadherin (E-cad), -smooth muscle actin (α-SMA), β-catenin, phosphorylated Smad3, and NGAL in control, IRI-treated and IRI/PD173074-treated mice. (c) Quantification of the data in (b). (*P < 0.05; **P < 0.01). (d) Average concentrations of serum FGF-23 in mice (mean ± SD; n = 6 mice per group; **P < 0.01)

To further determine how FGF-23 promotes renal fibrosis, we treated IRI mice daily with the pan-FGFR inhibitor PD173074 for 2 weeks beginning on the first day after surgery. The serum levels of FGF-23 were increased after IRI, and there was no significant difference between IRI and mice treated with PD173074 (Fig. 4d). The kidney histologic examinations showed that PD173074 mitigated renal fibrosis induced by IRI (Fig. 4a). PD173074 mitigated the abnormal expression of neutrophil gelatinase-associated lipocalin (NGAL), β-catenin, E-cadherin, α-SMA, and phosphorylated Smad3, indicating that PD173074 can protect the kidney from previous damage and functional abnormalities induced by IRI (Fig. 4b, c). PD173074 treatment attenuates creatinine and urea nitrogen in IRI mice (Supplementary Fig. 1).

## Discussion

AKI is an increasing health burden with high morbidity and mortality rates worldwide. AKI is a risk factor for CKD development. The understanding of AKI developing into CKD was derived from retrospective clinical observations. AKI is now considered to be an independent risk factor for the development of CKD [17]. However, there are limited experimental data directly addressing the AKI-to-CKD transition. Thus, we explored the potential profibrotic role of FGF-23 after AKI and described its molecular mechanism.

Bone is the major source of circulating FGF-23. FGF-23 acts on the kidneys through fibroblast growth factor receptors (FGFRs) and the coreceptor Klotho to promote phosphaturia by downregulating phosphate transporters, as well as controlling vitamin D metabolizing enzymes to reduce blood 1,25-dihydroxyvitamin D [18]. Intact FGF-23 (iFGF-23) can be cleaved into N-terminal FGF-23 and C-terminal FGF-23 (cFGF-23) by Furin or plasminogen activators [19]. According to the literature, circulating FGF-23 is nearly all iFGF-23 in haemodialysis patients due to impaired FGF-23 cleavage [20], and approximately 80% of circulating FGF-23 is iFGF-23 in predialysis patients [21]. In this study, we selected iFGF-23 concentration detection in human and mouse serum. Several studies have shown that when present in excess, FGF-23 can produce off-target effects beyond classical endocrine mineral processing. This effect is thought to occur in the hearts of CKD patients, possibly driving hypertrophic and fibrotic signalling programs through the inappropriate activation of cells that do not express their physiological coreceptor Klotho [22]. In our previous study, we demonstrated that FGF-23 was elevated in the serum of CKD rats, which led to myocardial hypertrophy and promoted cardiomyocyte fibrosis [23]. Most notably, it has been shown that FGF-23 enhances the profibrotic signalling cascade in injury-induced renal fibroblasts by activating FGFR4 and upregulating the calcium transporter transient receptor potential cation channel 6 [24–26].

In this study, we found that circulating FGF-23 levels were significantly increased in AKI patients, and FGF-23 levels in AKI patients were closely related to the recovery of renal function; in other words, the higher the FGF-23 level was, the higher the possibility of progression to CKD (Fig. 1). Moreover, after ischaemia‒reperfusion, continuous increases in serum FGF-23 were observed in IRI mice (Fig. 2). Based on these results, we hypothesized that FGF-23 could mediate CKD progression.

Several pathways are thought to be involved in FGF-23-mediated promotion of CKD. For instance, hyperphosphatemia is closely related to the degree of tubulointerstitial damage [26]. FGF23 is associated with the inflammatory response [27], endothelial injury [22], and sympathetic and RAAS activation [28]. Most notably, renal fibrosis is the pathological basis of CKD. In recent years, studies have revealed that the canonical TGF-β1/smad3 signalling pathway plays an important role in the progression of renal fibrosis, and persistent activation of the Wnt/β-catenin pathway is involved in promoting the development of AKI to CKD. Coincidently, our previous study showed that TGF-β and Wnt/β-catenin pathway inhibition could reverse renal tubular fibrosis [29]. According to these results, we hypothesized that FGF-23 accelerated AKI-CKD via the TGF-β and Wnt/β-catenin pathways. Through in vivo and in vitro experiments, we suggest that FGF-23 could not only lead to renal tubular fibrosis directly but could also activate the fibrotic TGFβ/Smad and Wnt/β-catenin pathways (Figs. 2 and 3). In addition, PD173074, a blocker of FGFR, inhibited this effect both in vivo and in vitro. (Figures 2 and 4). On the other hand, myofibroblasts are the main source of extracellular matrix (ECM) during kidney fibrosis [30], and genetic fate-tracing data in mice and histological analyses of human tissue suggested that epithelial, endothelial, haematopoietic and resident mesenchymal cells all contribute to fibrosis [31]. Does FGF-23 lead to myofibroblast activation and eventually cause renal fibrosis by damaging renal tubular epithelial cells? This needs to be confirmed by further studies.

In summary, we extended our understanding of FGF-23 in AKI-CKD. We showed a sustained increase in circulating FGF-23 in AKI patients and IRI mice, which may be related to AKI progression to CKD. We further confirmed that the upregulation of FGF-23 facilitates the activation of TGF-β and Wnt/β-catenin signalling. Moreover, FGF-23 upregulation may result in renal fibrosis via TGF-β and Wnt/β-catenin activation. We believe these data not only uncover a novel mechanism of AKI-CKD but also hint at a potential therapeutic target for AKI treatment.

## Electronic supplementary material

Below is the link to the electronic supplementary material.

Supplementary Material 1

Supplementary Material 2

## Data Availability

The datasets used and analysed during the current study are available via corresponding author on reasonable request.
