# Supplementary material for: A potential link between fibroblast growth factor-23 and the progression of AKI to CKD
Source: BMC Nephrol. 2023 Apr 4;24:87. doi: 10.1186/s12882-023-03125-1 (PMC10074805; doi:10.1186/s12882-023-03125-1)
Supplement: Supplementary file 1 — Supplementary Material 1 [file 12882_2023_3125_MOESM1_ESM.docx]

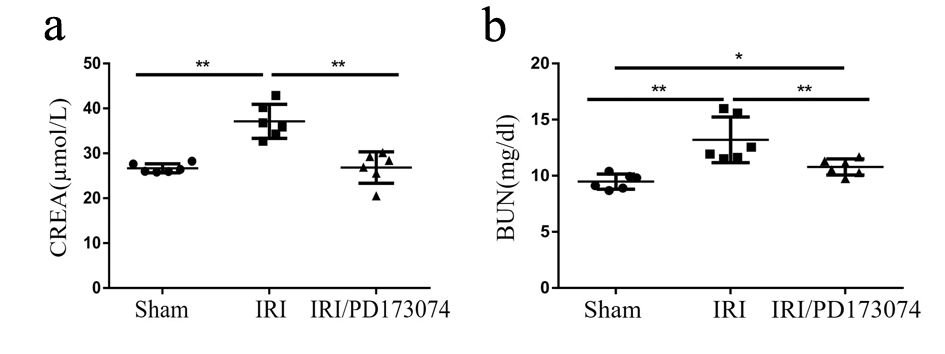


Supplementary Fig 1. Levels of creatinine and BUN in the serum of each group of mice (mean ± SD; n = 6 mice per group; *P<0.05; **P<0.01).

Supplementary Table 1. Logistic regression analysis of the correlation of FGF-23 with AKI

| Variable | FGF-23 | |
| --- | --- | --- |
|  | OR | *P* |
| AKI (yes/not) | 1.044 | <0.001 |

Supplementary Table 2. Logistic regression analysis of the correlation of FGF-23 with AKI post CKD

| Variable | FGF-23 | | |
| --- | --- | --- | --- |
|  | OR | | *P* |
| AKI post CKD (yes/not) | | 1.018 | 0.018 |

AKI post CKD: AKI progresses to CKD.
