# Supplementary material for: A potential link between fibroblast growth factor-23 and the progression of AKI to CKD
Source: BMC Nephrol. 2023 Apr 4;24:87. doi: 10.1186/s12882-023-03125-1 (PMC10074805; doi:10.1186/s12882-023-03125-1)
Supplement: Supplementary file 2 — Supplementary Material 2 [file 12882_2023_3125_MOESM2_ESM.docx]

**The full and unedited version of the original western gels**


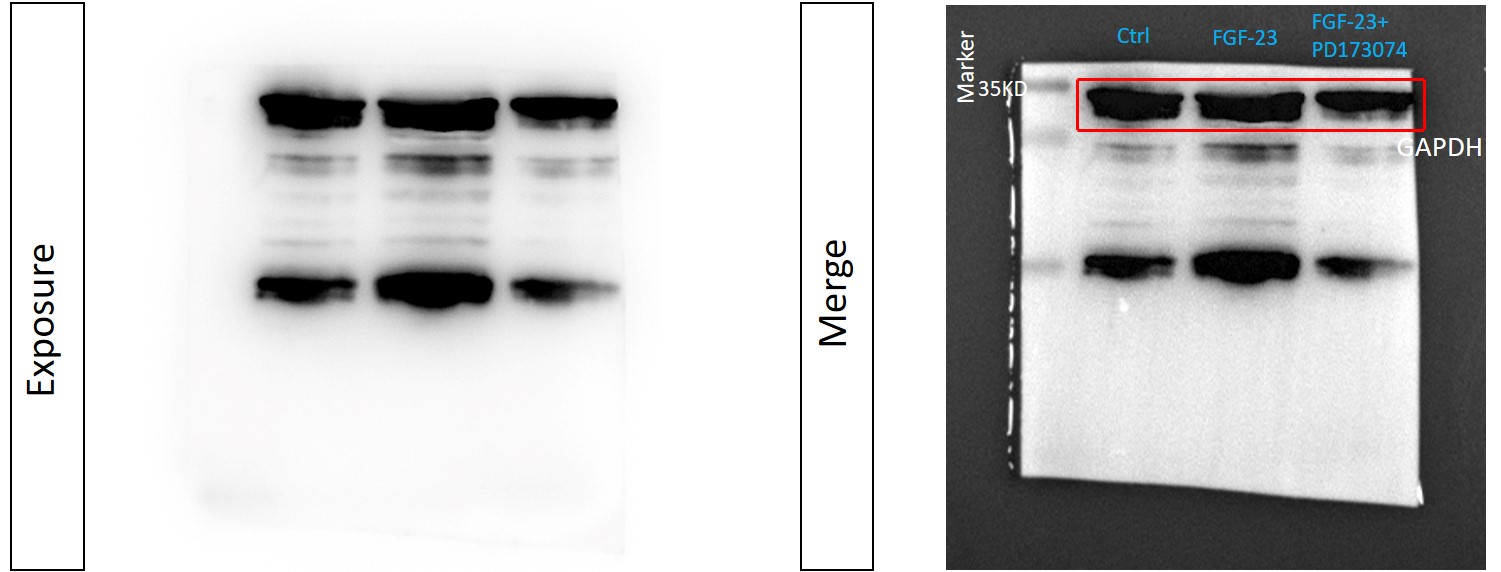


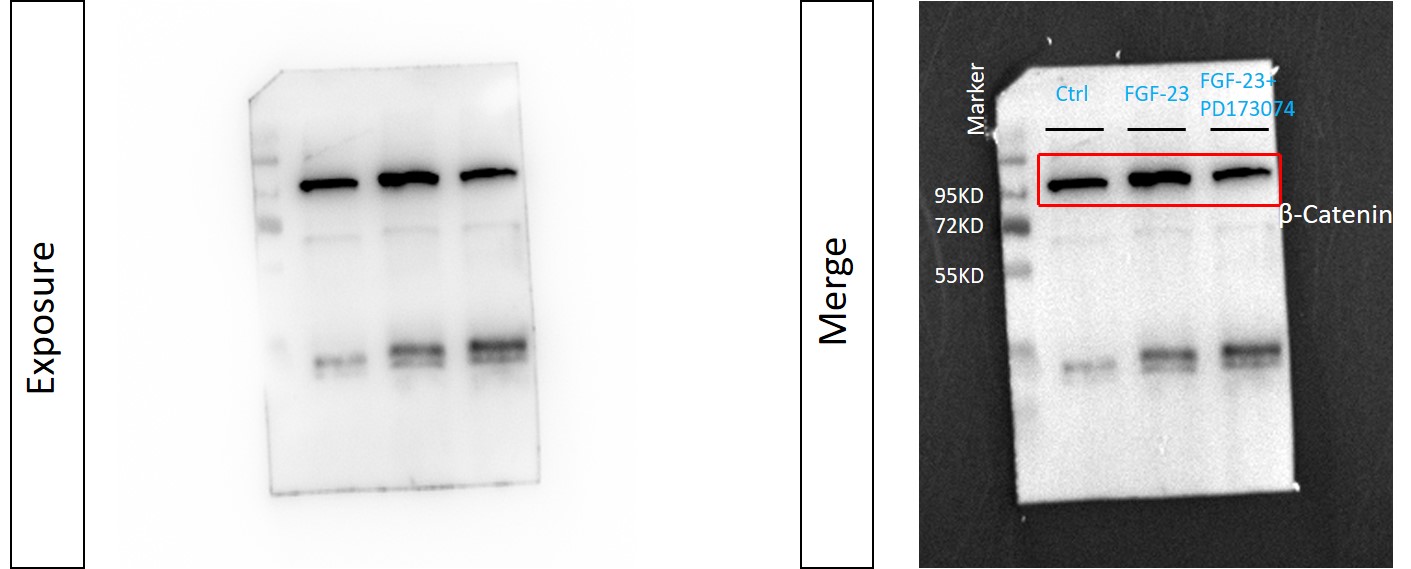


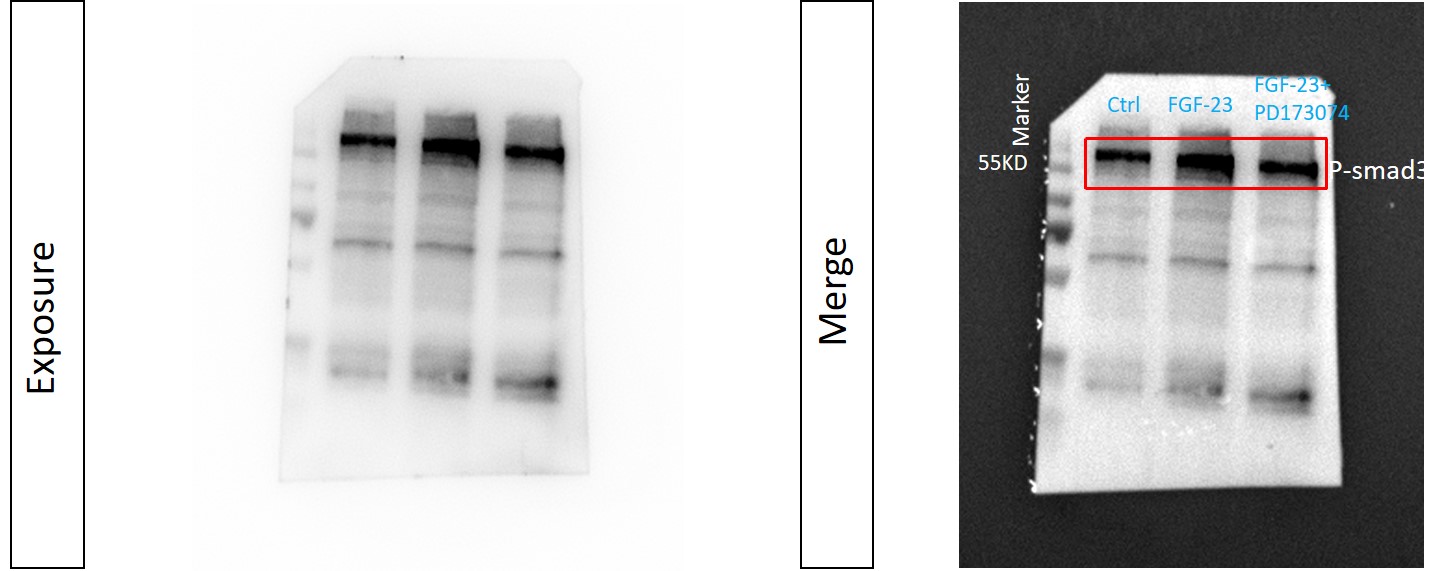


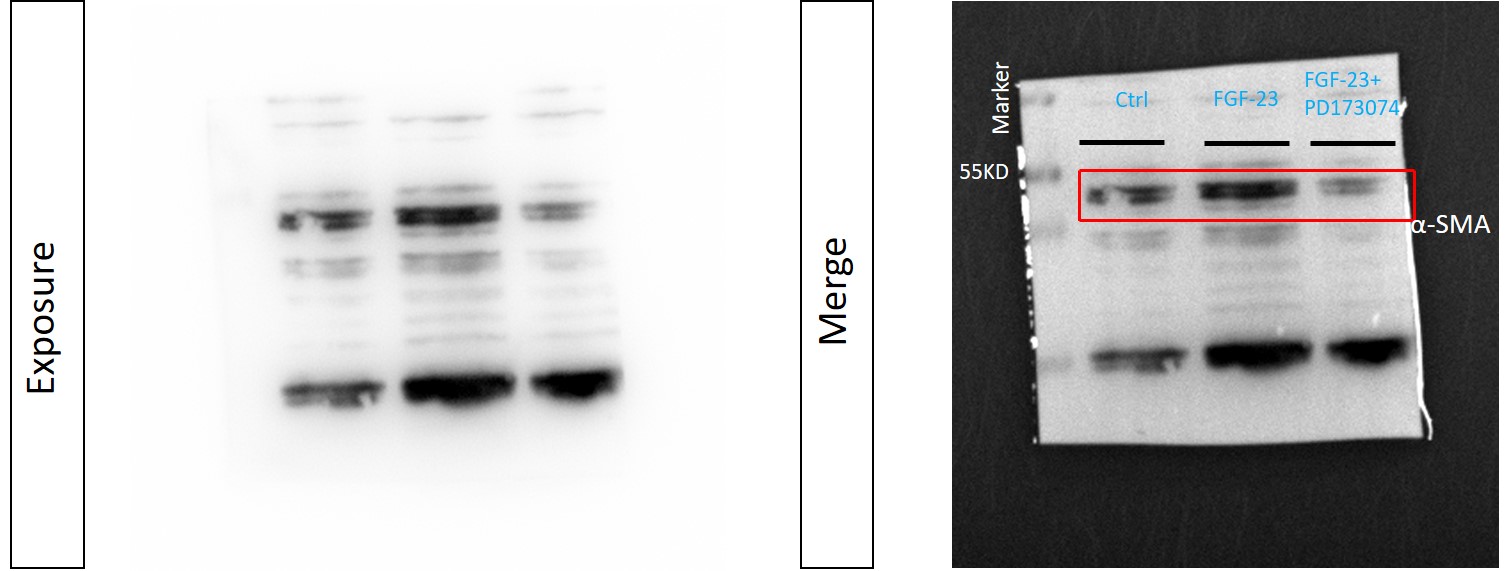


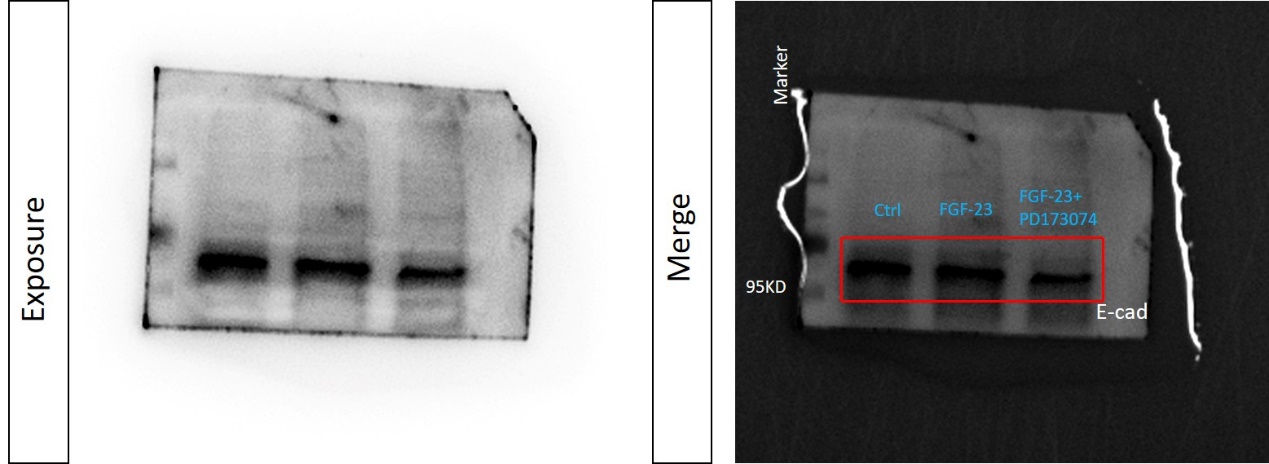


Full unedited gel for Figure 3


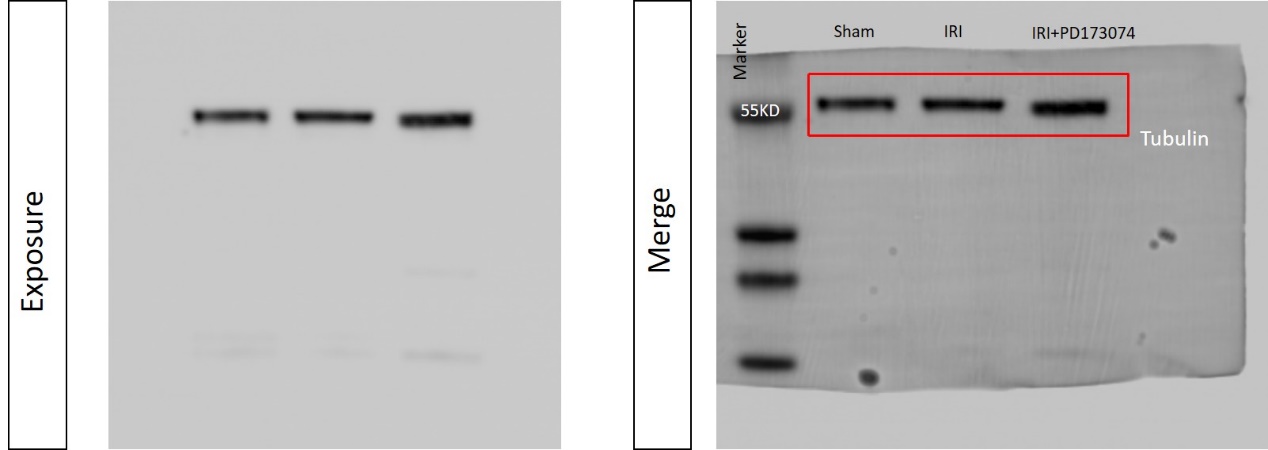


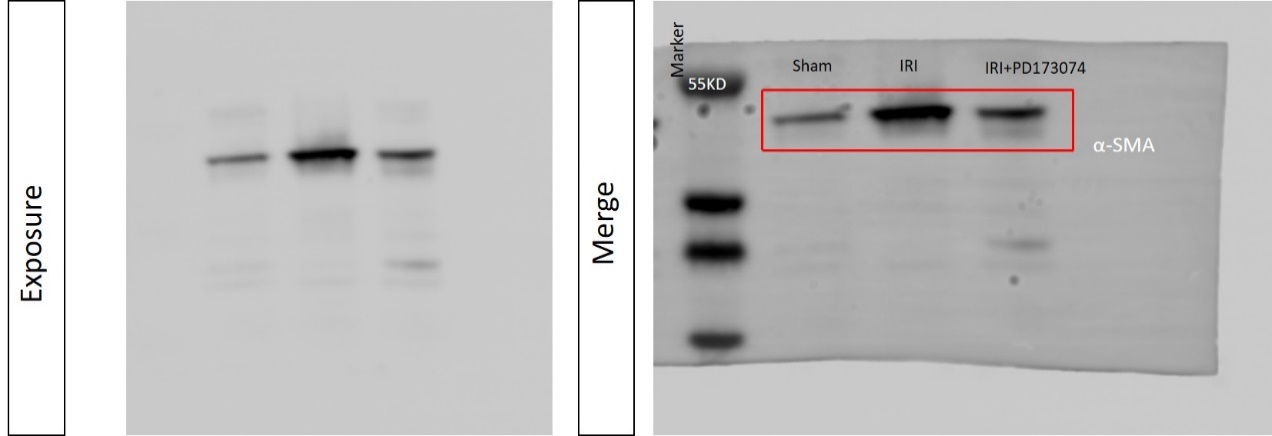


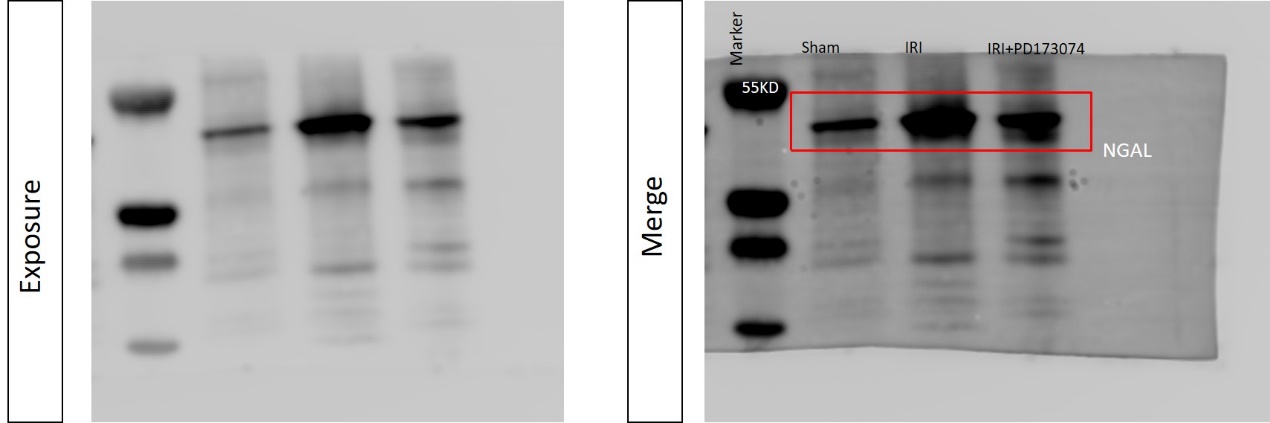


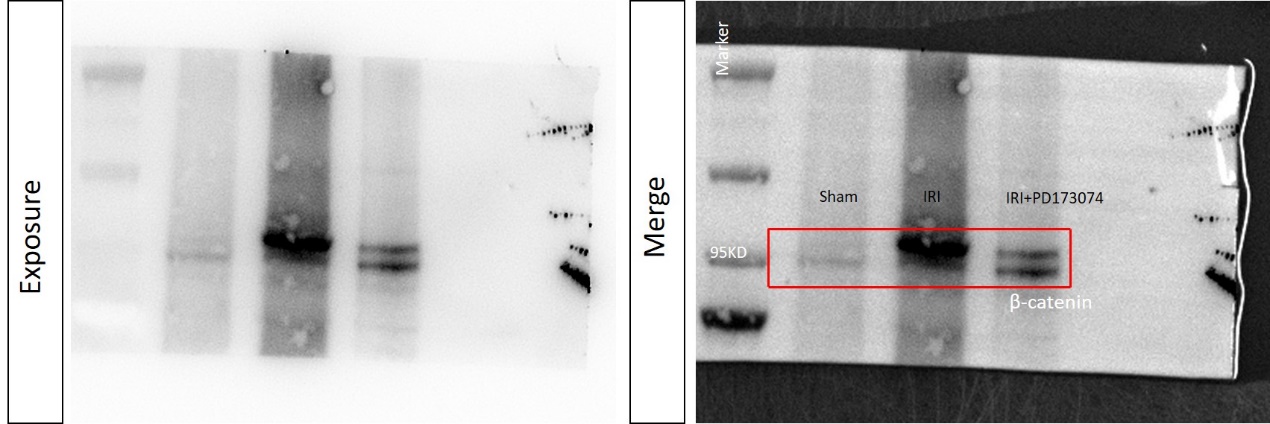


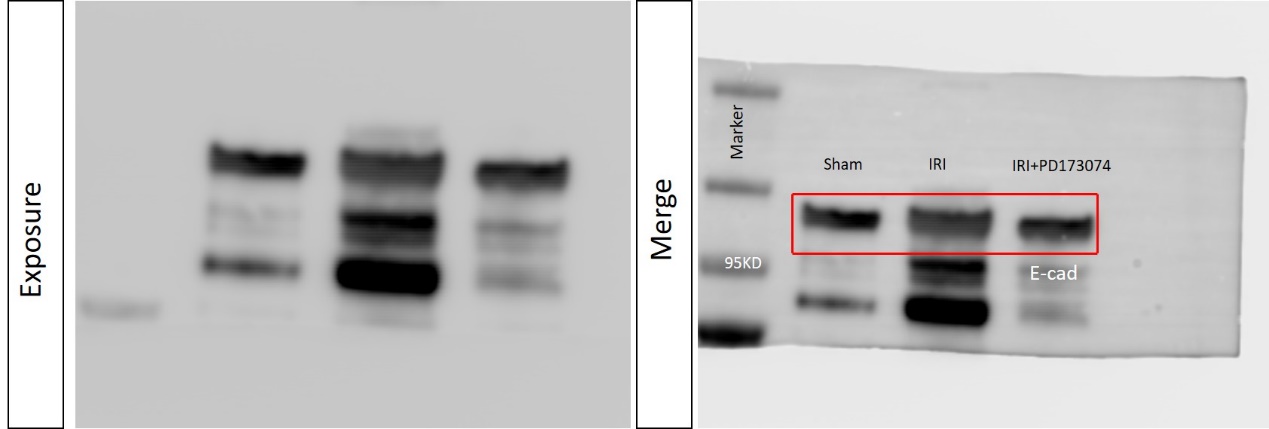


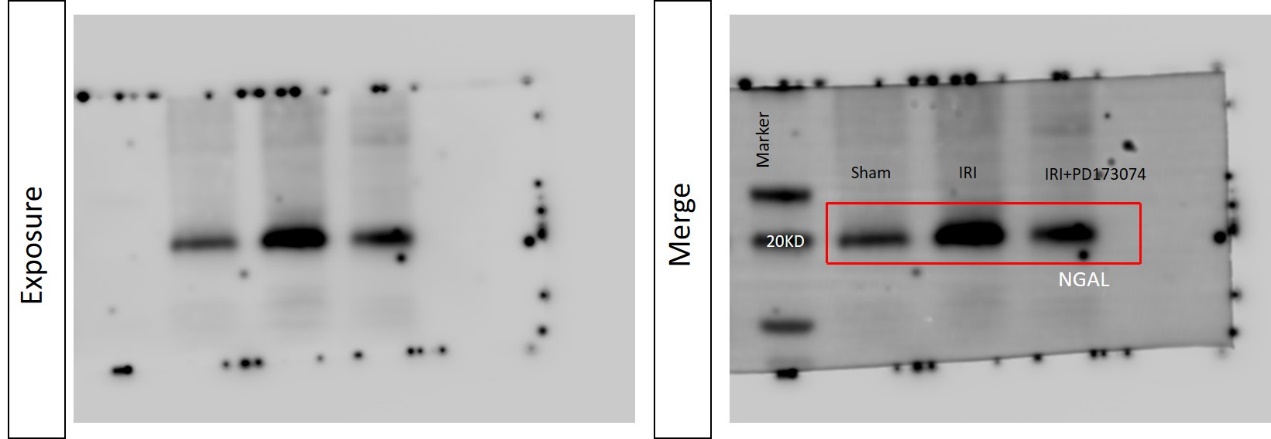


Full unedited gel for Figure 4
